# Circadian Genes, *xBmal1* and *xNocturnin*, Modulate the Timing and Differentiation of Somites in *Xenopus laevis*

**DOI:** 10.1371/journal.pone.0108266

**Published:** 2014-09-19

**Authors:** Kristen L. Curran, Latoya Allen, Brittany Bronson Porter, Joseph Dodge, Chelsea Lope, Gail Willadsen, Rachel Fisher, Nicole Johnson, Elizabeth Campbell, Brett VonBergen, Devon Winfrey, Morgan Hadley, Thomas Kerndt

**Affiliations:** University of Wisconsin-Whitewater, Department of Biological Sciences, Whitewater, Wisconsin, United States of America; University of Texas Southwestern Medical Center, United States of America

## Abstract

We have been investigating whether *xBmal1* and *xNocturnin* play a role in somitogenesis, a cyclic developmental process with an ultradian period. Previous work from our lab shows that circadian genes (*xPeriod*1, *xPeriod2*, *xBmal1*, and *xNocturnin*) are expressed in developing somites. Somites eventually form the vertebrae, muscles of the back, and dermis. In *Xenopus*, a pair of somites is formed about every 50 minutes from anterior to posterior. We were intrigued by the co-localization of circadian genes in an embryonic tissue known to be regulated by an ultradian clock. Cyclic expression of genes involved in *Notch* signaling has been implicated in the somite clock. Disruption of *Notch* signaling in humans has been linked to skeletal defects in the vertebral column. We found that both depletion (morpholino) and overexpression (mRNA) of xBMAL1 protein (bHLH transcription factor) or xNOCTURNIN protein (deadenylase) on one side of the developing embryo led to a significant decrease in somite number with respect to the untreated side (p<0.001). These manipulations also significantly affect expression of a somite clock component (*xESR9*; p<0.05). We observed opposing effects on somite size. Depletion of xBMAL1 or xNOCTURNIN caused a statistically significant decrease in somite area (quantified using NIH ImageJ; p<0.002), while overexpression of these proteins caused a significant dose dependent increase in somite area (p<0.02; p<0.001, respectively). We speculate that circadian genes may play two separate roles during somitogenesis. Depletion and overexpression of xBMAL1 and NOCTURNIN both decrease somite number and influence expression of a somite clock component, suggesting that these proteins may modulate the timing of the somite clock in the undifferentiated presomitic mesoderm. The dosage dependent effects on somite area suggest that xBMAL1 and xNOCTURNIN may also act during somite differentiation to promote myogenesis.

## Introduction

In vertebrate embryos, somites are blocks of paraxial mesoderm that form on either side of the neural tube from the presomitic mesoderm. Somites will later form the muscles of the back, dermis, and parts of the vertebra. As the embryo elongates, cells are added to the posterior region of the presomitic mesoderm from a population of stem cells in the tailbud, while pairs of somites bud off from the anterior edge of the presomitic mesoderm. Somite formation has been shown to be a developmentally timed event. In *Xenopus*, pairs of somites form about every 50 minutes, extrapolated from [1], [2], while somite formation occurs every 90 minutes in chick [3], 30 minutes in zebrafish and 120 minutes in mouse [4]. The mechanism for the timing of somitogenesis has been described using the clock-wavefront model. The molecular mechanism that regulates the formation of somites includes members of the Notch, WNT, and FGF gene families [5]. Each cell in the presomitic mesoderm contains an autonomous oscillator that is synchronized with its cohorts via Notch-Delta signaling [6], [7], [8]. At the anterior border of the presomitic mesoderm oscillation within each cell stops, allowing differentiation of the tissue into a new pair of somites [9]. The wavefront refers to the region of the presomitic mesoderm where somites form and the clock stops [10], [11], [12]. The mechanism of molecular time-keeping involves maintenance of a balance of bHLH transcriptional co-repressors of the Notch family (*Her1, Hes 6, Hes7*) that control somite clock components [13]. Mathematical modeling supported by experimental evidence suggests that delayed coupling between cells of the presomitic mesoderm can influence the period of somite formation and affect the number and size of somites formed [14], [15]. Recent experiments in chick showed that multiple ectopic somites can form simultaneously from presomitic mesoderm treated with noggin. In these experiments no obvious oscillations of somite clock components was observed [16]. Therefore, the somite clock may act to direct where and when somites will form in the presomitic mesoderm, but may not be required for somite differentiation.

The molecular time-keeping mechanism for maintaining a cell autonomous circadian rhythm is well defined and also involves a delayed negative transcription/translation feedback loop. In vertebrates (mouse specifically), the level of mRNA from three relatives of the *Drosophila Period* gene (*mPer1*, *2* and *3*) begins to rise at slightly different times during the day and the mRNA is translated into protein. The increase in *mPer* RNA is due to transcriptional activation by a heterodimer consisting of CLOCK and BMAL1 [17], [18], [19], [20], [21]. *mPer 1–3* along with *Cryptochromes* (*Cry1* and *Cry2*) inhibit transcription activation by the CLOCK/BMAL1 heterodimer [22], [23]. This core transcription/translation feedback loop maintains, approximately, a 24 hour period as well as influencing the rhythmic expression of downstream targets called clock controlled genes (CCGs) [24], [25], [26]. One CCG of interest, *xNocturnin*, is a deadenylase whose transcription peaks during the night [27], [28], [29], [30]. Deletion of *Nocturnin* in mice influences sensitivity to glucose and insulin, storage of lipid, and absorption of nutrients [31], [32], [33]. Characterization of circadian gene expression during *Xenopus* development reveals that three circadian oscillator genes (*xPeriod1, xPeriod2,* and *xBmal1*) and one CCG (*xNocturnin*) are expressed in the developing somites, although not always within the same region of a somite [34]. Somitogenesis begins in *Xenopus* around stage 17, 18.75 hours post fertilization and we have shown that circadian genes are expressed in developing somites by at least stage 25 (24 hours post fertilization) [2], [34]. Therefore, we were interested in pursuing the hypothesis that circadian genes may play a role in the process of somitogenesis.

Comparison of independent microarray analyses performed in mouse show that genes involved in the circadian and somite molecular clocks display both circadian and ultradian rhythms in adult and embryonic tissues. Microarray analysis during mouse somitogenesis uncovered ultradian rhythms of circadian genes, between 30 and 156 minutes, similar to somite clock genes (Table 1) [35]. Circadian expression of somite clock genes can occur in the adult mouse suprachiasmatic nucleus (SCN), liver, and heart [36], [37], [38], [39]. Specifically, Notch family members *Hes1*, *Hes5*, *Id*, *Notch*, and *Delta* display a putative circadian rhythm as well as WNT family members *Axin2*, *GSK3*, *Dkk1*, *Sp5*, and *Tnfrsf9* (Table 1). Taken together these studies indicate that members of all three gene families can display both circadian and ultradian periods of expression suggesting that crosstalk could exist between gene products of the circadian and somite clocks.

**Table 1:** A compilation of results from microarray analyses of temporal expression of Notch, WNT, and circadian genes in the somites [34] and suprachiasmatic nucleus, liver, and heart [35], [36], [37], [38].

Gene Family	Gene name	Circadian (peak)	Somite Period (min.)
**Notch**			
	*Hes1*	Night	94
	*Hes5 (xESR9)*	Day	102
	*Id1*	Night	87
	*Notch*	Day	None
	*Delta*	Night (Heart)	52.5
**WNT**			
	*Axin2*	Night	102
	*Dkk1*	Day	112
	*Sp5*	Night	112
	*Tnfrsf9*	Night	94
**Circadian**			
	*Clock*	Constitutive in SCN Day in liver	30
	*Casein Kinase ε*	Constitutive	156
	CREB-P	Night*	34
	*Rora*	Day	71
	*Arnt1 (Bmal1 homologue)*	Night	32
	*Cry2*	Night	30

*CREB1 phosphorylation rhythm in *Xenopus* retina [42].

We initially framed our approach for investigating the role of circadian genes in somitogenesis using two alternate hypotheses. Since heterodimers of BMAL/CLOCK regulate *MyoD* expression in adult skeletal muscle [40], [41] we hypothesized that circadian gene products affect somite differentiation and myogenesis (skeletal muscle formation). Alternatively, circadian genes could be a component of the somite clock and influence the developmental timing of somitogenesis. Both *xBmal1* and *xNocturnin* have been shown to influence gene expression albeit in different ways and are highly expressed in the developing somites [34]. In this manuscript we show that three central oscillator genes, *xClock, xCryptochrome1* and *xCryprochrome2*, are also expressed within the developing somite. We report that depletion and overexpression of a central oscillator gene (*xBmal1*) and a CCG (*xNocturnin*) can affect somitogenesis in *Xenopus laevis*. Specifically, depletion of xBMAL1 or xNOCTURNIN protein decreased somite area while overexpression increased somite area. Surprisingly, depletion or overexpression of either protein led to a decrease in somite number but could also affect *xESR9* expression (a readout of somite clock timing; *Hes5* homologue) [42]. Our results suggest that xBMAL1 and xNOCTURNIN may play two separable roles during somitogenesis, the first is to modulate the timing of somitogenesis in the presomitic mesoderm and later influence the differentiation of somites.

## Results

### Revisiting circadian gene expression in the somites

**Figure 1:**
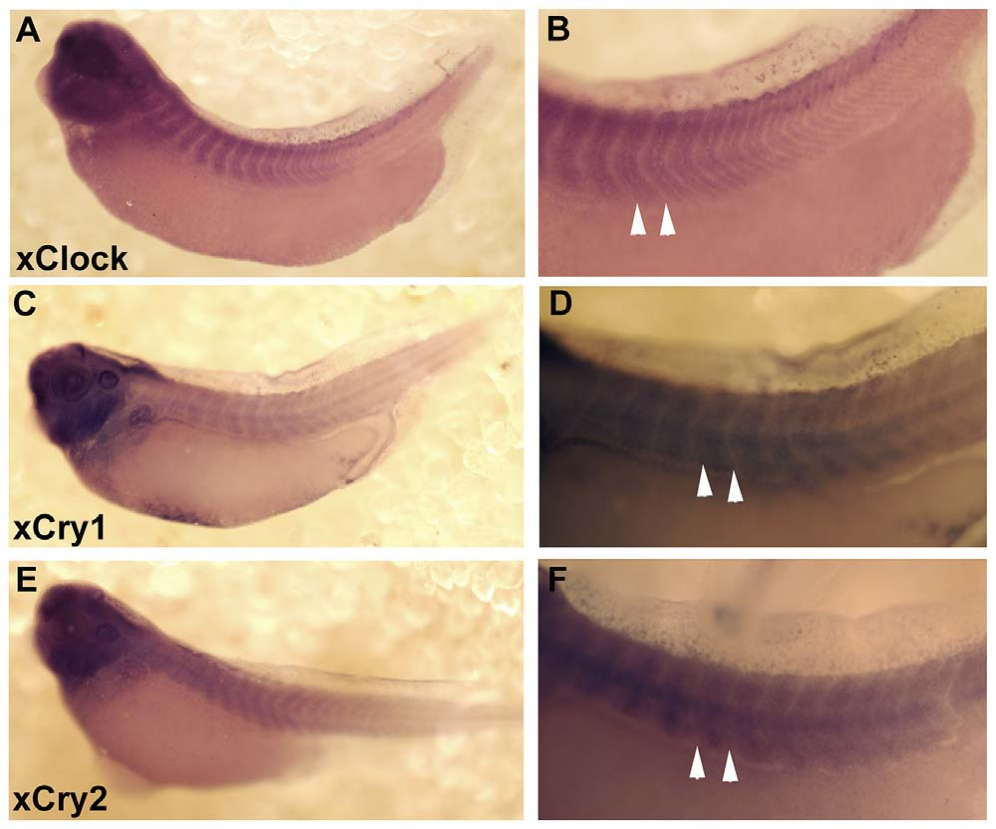
*xClock*, *xCry1*, and *xCry2* are expressed in developing somites of tailbud stage embryos. Expression of each gene in the whole embryo and somites is provided. Panels A and B show *xClock* expression in the developing somites of a stage 35/36 embryo. Panels C and D show *xCry1* expression in the developing somites of a stage 37/38 embryo. Panels E and F show *xCry2* expression in a stage 37/38 embryo. White arrow heads indicate the anterior-posterior borders at the ventral extent of one somite.

We have previously published the developmental expression *of xPeriod1*, *xPeriod2*, *xBmal1*, and *xNocturnin* and showed differential expression pattern of these genes within the somites at stage 40 (56 hours post fertilization, hpf). Here we extend this analysis to show that three additional central oscillator genes are also expressed in the developing somites. *xClock*, *xCryptochrome 1* (*xCry1*), and *xCryptochrome 2* (*xCry2*) are present in the developing somites of tailbud stage embryos (Figure 1). In figure S1 we show the co-localization of *xBmal1, xNocturnin, xPeriod1, xCry1, xCry2*, and *xClock* with a somite marker (12/101) in stage 35–38 embryos [43]. The developmental expression of *xCry1*, *xCry2*, and *xClock* is characterized in Figure S2. In general *xCry1*, *xCry2*, and *xClock* were detected first in the developing brain and spinal cord, followed by expression in the somites and various organs and tissues at distinct times during early development. The onset of expression in specific organs and tissues is different for each gene which is similar to our previous findings for *xPeriod1*, *xPeriod2*, *xBmal1*, and *xNocturnin* [34].

### Depletion of xBMAL1 and xNOCTURNIN protein decreased somite number and size

**Figure 2:**
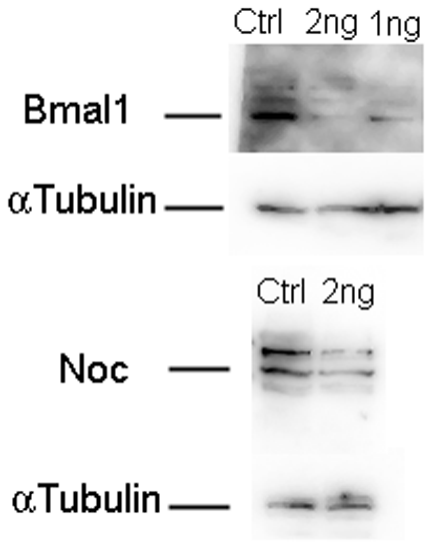
Reduction in xBMAL1 and xNOCTURNIN protein by morpholino injection. Both cells of a two celled embryos were injected with 1 ng Control Morpholino (Ctrl; 2 ng total), 1 ng or 500 pg of xBmal1 MO (Bmal1; 2 ng and 1 ng total) and 1 ng of *xNocturnin* MO (Noc; 2 ng total). Significant reduction of xBMAL1 protein (69Kd) was observed with injection of 2 ng or 1 ng *xBmal1* MO compared to control MO injection (0.11 and 0.26 relative to control MO injected protein levels). An approximate 50% reduction of NOCTURNIN (43Kd, indicated) protein was observed when embryos were injected with a total of 1 ng *xNocturnin* MO (0.49 relative to control MO injected protein levels). The Nocturnin antibody also recognizes a larger (62Kd) band which likely represents a postranslationally modified form of xNOCTURNIN [28]. Alpha tubulin (100Kd) was used as a loading control for each lane.

Our initial analyses of the developmental expression of circadian genes showed that the two most highly expressed genes in the somites were *xBmal1* and *xNocturnin* [34]. Therefore, we tested whether depletion of xBMAL1 or xNOCTURNIN protein could affect somitogenesis during early *Xenopus laevis* development. To test this hypothesis we injected control morpholino (MO; 1.5 ng, 1 ng, or 500 pg), *xBmal1* MO (1 ng or 500 pg), or *xNocturnin* MO (1.5 ng, 1 ng, 500 pg) into one cell of a 2 cell embryo. Injected embryos were cultured to stage 25–28 and analyzed by *in situ* hybridization for a marker of somitogenesis timing (*xESR9*) [42] followed by whole mount immunohistochemistry using 12/101 (somite/muscle marker) [43]. The specificity of each morpholino was confirmed by western blot. Each cell of two cell embryos was injected with 1 ng or 500 pg of *xBmal1* MO or 1 ng of *xNocturnin* MO. The embryos were then cultured to stage 25 and prepared for western blot analysis. The *xBmal1* MO caused a dramatic decrease in the amount of detectable xBMAL1 protein (2 ng/embryo, 89% reduction; 1 ng/embryo, 74% reduction) while the *xNocturnin* MO (2 ng/embryo) caused a 49% reduction in xNOCTURNIN protein with respect to the embryos injected with control MO (Figure 2).

**Figure 3:**
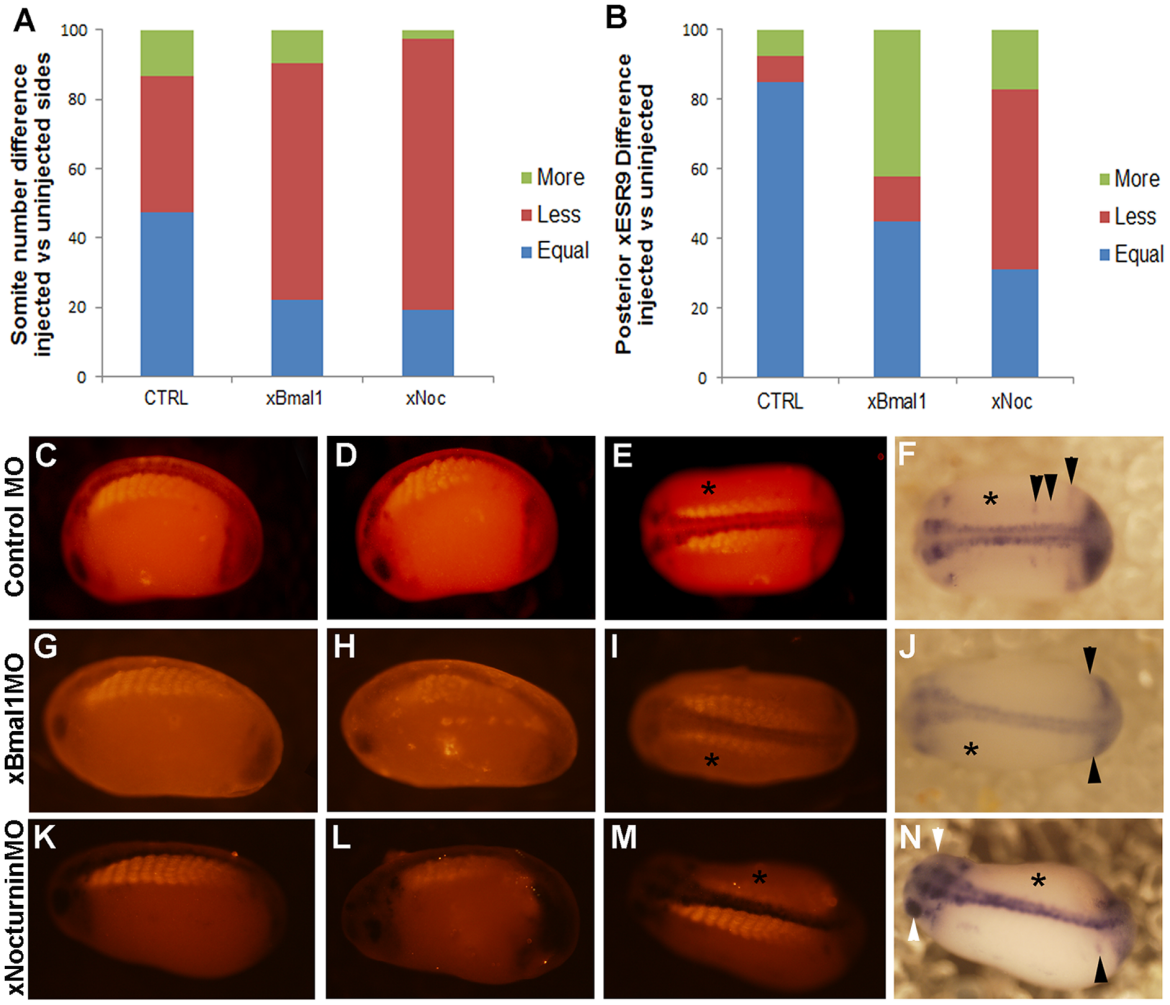
Depletion of xBMAL1 or xNOCTURNIN results in fewer somites on the injected side (asterisk). Results of injection of 1 ng of either control, *xBmal1*, or *xNocturnin* MO are shown. Panel A shows the percent of embryos with equal, less, or more somites on the injected side when compared to the uninjected side. Embryos were also analyzed for effects on the posterior striping pattern of *xESR9* (B). The percent of embryos with equal, less, or more *xESR9* stripes on the injected side when compared to the uninjected side is indicated on the vertical axis while the type of MO is shown on the horizontal axis. All pictures shown in panels C-N are displayed with anterior to the left. Panels C, G, and K display the uninjected side for each treatment. Panels D, H, and L display the injected side for control MO, *xBmal1*MO, and *xNocturnin*MO, respectively. Panels E, I, and M show a dorsal view of each embryo for somite staining while panels F,J, and N show a dorsal view of *xESR9* expression. Black arrowheads in F show normal *xESR9* expression in the posterior. Arrowheads in J show an example where no stripes are visible but the posterior border was different between injected and uninjected sides of the embryo. The embryo in panel N experienced slight exogastrulation, but somite expression and *xESR9* expression were evaluated. White arrowheads show an example of decreased expression of *xESR9* in the eye on the side injected with *xNocturnin* MO.

Depletion of xBMAL1 or xNOCTURNIN protein consistently caused fewer somites to form on the injected side when compared to control MO injection (Figure 3; Figure S3; Table 2). Analysis consisted of counting the number of somites on the injected and uninjected side of each embryo. Next, the difference in somite number between the injected and uninjected sides was calculated and the percentage of embryos with equal, less, or more somites on the injected side was determined. The proportion of embryos with each score is shown in Figure 3A for 1 ng injections only. ANOVA analysis showed a significant difference between injection conditions (p<0.001). A posthoc LSD test comparing experimental MO injection to control MO shows a significant difference in effects on somite number in embryos injected with *xBmal1* MO (p<0.006) and *xNocturnin* MO (p<0.001). Control MO injection could decrease somite number but these effects were variable. In contrast, injection of *xBmal1* MO and *xNocturnin* MO caused consistent and in many cases dramatic (33.3% of embryos; Table 2) decrease in somite number (Figure 3A, Figure S3, Table 2). Typical examples of the effects of MO injection are presented in Figure 3 showing the uninjected side of each embryo (C, G, K), followed by the injected and dorsal view for 1 ng control MO (Figure 3 D,E), 1 ng *xBmal1* MO (Figure 3 H,I), and 1 ng *xNocturnin* MO (Figure 3 L,M).

**Table 2:** Effects of morpholino injection on somite number in stage 24–28 embryos.

Somite number (injected vs uninjected)	CTRLMO (1 ng) N = 38	*xBmal1* MO (1 ng) N = 54	*xNocturnin* MO (1 ng) N = 42
Equal	47.4%	22.2%	19%
Decreased by 1–2 somites	39.5%	35.2%	50%
Decreased by less than 2	0%	33.3%	28.6%
Increased by 1–2 somites	2.6%	9.3%	2.4%
Increased by more than 2	10.5%	0%	0%

**Figure 4:**
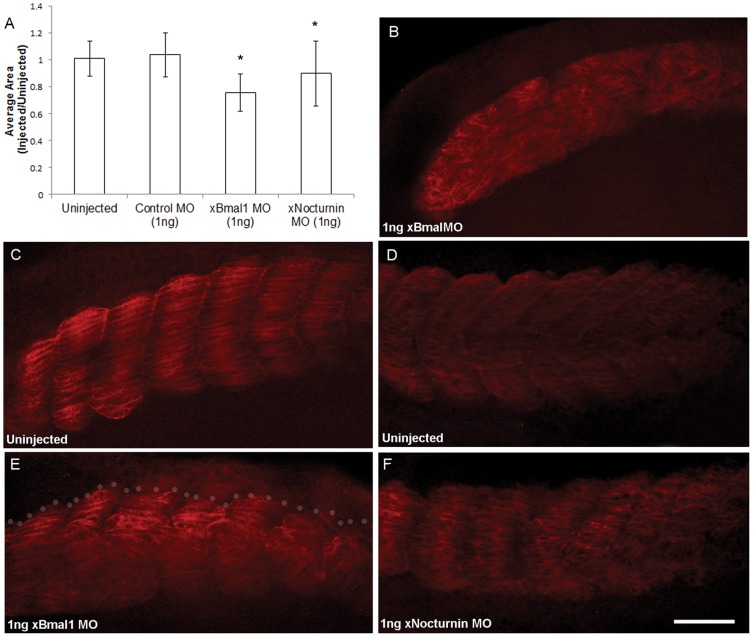
Depletion of xBMAL1 or xNOCTURNIN results in smaller, disorganized somites with disrupted somite boundaries. Panel A represents the results of analyzing the somite area (uninjected side vs injected) of pairs of somites using NIH Image J. The average ratio of the area of injected to uninjected sides of the embryos analyzed is shown with error bars representing standard deviation. Somites injected with either *xBmal1* MO (1 ng) or *xNocturnin* MO (1 ng) showed a significant decrease in somite area (*) when compared to the control MO (1 ng). Images in panels B-F are oriented with the anterior of the embryo to the left and dorsal up. Panel B shows an extreme phenotype where somite boundaries have been eliminated on the injected side (1 ng *xBmal1* MO). Panels C (uninjected) and E (injected) show effects on somite size, organization and boundaries in an embryo injected with 1 ng (*xBmal1* MO). Expression from the uninjected side of the embryo can be seen through the cleared embryo in E, grey dots separate foreground image from the background. Panels D and F show similar results were observed with injection of *xNocturnin* MO (1 ng) although there is usually less disruption of somite borders (D, uninjected; F, *xNocturnin* MO injected). All images were taken at the same magnification (scale bar = 10 µm).

Depletion of xBMAL1 or xNOCTURNIN protein also resulted in decreased somite area and affected somite boundary formation and organization. We initially observed that somites of *xBmal1* or *xNocturnin* MO injected embryos appeared smaller on the injected side. To confirm this observation we took pictures of the left and right sides of control MO, *xBmal1* MO, and *xNocturnin* MO injected embryos and compared the area of muscle actin expression for each somite pair (somite 3 from injected side vs somite 3 on the uninjected side) using NIH ImageJ [44]. The ratio of injected to uninjected areas was calculated for three consecutive somite pairs and an average of these ratios was reported for each embryo. The side views of the uninjected and injected sides of each embryo in figure 3 are examples of the type of image analyzed using NIH Image J [44]. ANOVA analysis resulted in a significant difference in injection condition (p<0.002). Next a posthoc LSD test was used to compare the average somite areas of embryos injected with *xBmal1* or *xNocturnin* MO injection with control MO injected embryos. The results of this analysis showed a significant decrease in somite area between *xBmal1* MO (1 ng; N = 11; p<0.001) and *xNocturnin* MO (1 ng; N = 17; p<0.04) injected embryos when compared to control MO injection (1 ng; N = 18; Figure 4A).

Experimental MO injection resulted in less distinct boundaries between somites and the cells of the somite were less organized on the injected side. In extreme cases embryos injected with either MO (*xBmal1* or *xNocturnin*) displayed a streak of muscle actin staining on the injected side indicating failure to form boundaries (Figure 4B) or complete failure in somite formation indicated by absence of 12/101 staining. More typical observations of disrupted boundaries and disorganized somites are shown in confocal images of cleared embryos. Figure 4C (uninjected) and E (injected) were treated with 1 ng *xBmal1* MO while embryos shown in Figure 4D (uninjected) and F (injected) were treated with 1 ng *xNocturnin* MO. In general, depletion of xNOCTURNIN seemed to have less of an effect on somite organization and boundary formation than depletion of xBMAL1.

Depletion of xBMAL1 or xNOCTURNIN protein can also affect the expression of a marker of the somite clock oscillation (*xESR9*) in the presomitic mesoderm. Normally, *xESR9* is strongly expressed in the tailbud of stage 25–28 embryos. *xESR9* has been shown to oscillate in the presomitic mesoderm, detectable as stripes of expression that arise from the tailbud. These stripes presage somite formation (figure 3F, arrowheads) [42]. Embryos injected with either *xBmal1* or *xNocturnin* MO in one cell of a two cells showed an altered extent of expression in the tailbud as well as an altered pattern of stripe formation (Figure 3B). In many embryos, the posterior border of *xESR9* expression in the presomitic mesoderm was altered on the injected side when compared to the uninjected side (Figure 3J, arrowhead). Li and colleagues [42] report that *xESR9* can occasionally be expressed asynchronously in the left vs the right sides of the developing embryo which necessitates careful comparison of control MO injected embryos with experimental MO injection. *xESR9* expression was evaluated by calculating the difference in the number of visible stripes between the injected and uninjected sides. The proportion of embryos with less or more stripes on the injected side is shown in Figure 3B. Due to the cyclic nature of *xESR9* expression, the resulting data was nonparametric necessitating the use of the Mann-Whitney U test to evaluate whether *xESR9* was affected by injection. The expression of *xESR9* was significantly different in embryos injected with *xBmal1* MO (N = 38; p<0.02) or *xNocturnin* MO (N = 52; p<0.05) when compared to control MO injected embryos, suggesting that *xBmal1* and *xNocturnin* may influence the somite clock. *xBmal1* and *xNocturnin* are expressed in the developing nervous system and eyes [34], therefore it is not surprising that depletion of these proteins also affects expression of *xESR9* in the eye, brain, and spinal cord (Figure 3N, white arrowheads; Figure S4).

### Overexpression of xBMAL1 or xNOCTURNIN protein affects somite number, size, and organization

**Figure 5:**
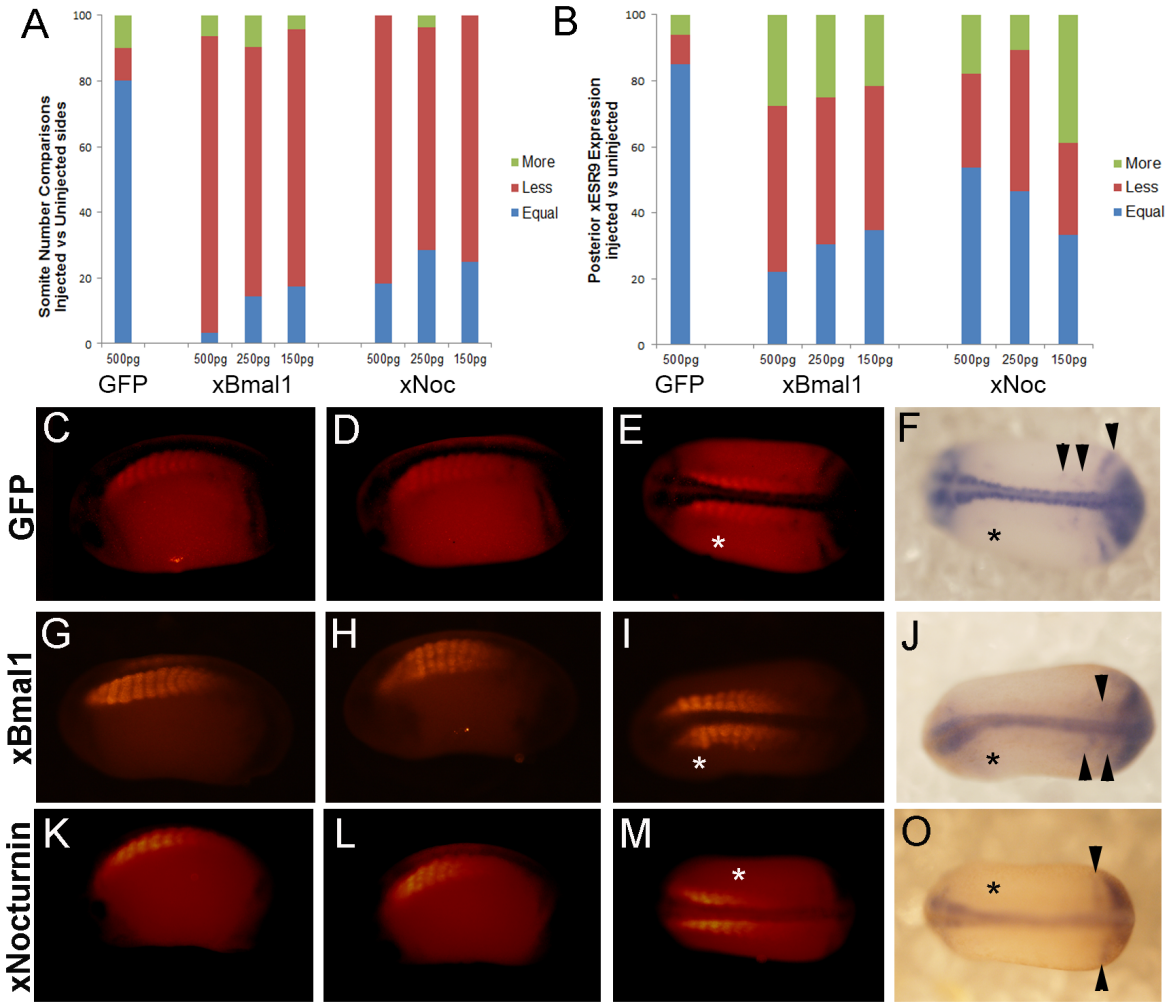
Overexpression of xBMAL1 or xNOCTURNIN results in fewer somites on the injected side (asterisk). Panel A shows the percent of embryos with equal, less, or more somites on the injected side when compared to the uninjected side. The concentration and type of RNA injected is shown on the horizontal axis. Embryos were also analyzed for effects on the posterior striping pattern of *xESR9* (B). The percent of embryos with equal, less, or more *xESR9* stripes on the injected side when compared to the uninjected side is indicated on the vertical axis while the concentration and type of RNA is shown on the horizontal axis. All pictures shown in panels C-O are displayed with anterior to the left and dorsal up. Panels C, G, K display the uninjected side for each treatment. Panels D, H, and L display the injected side. Panels E, I, and M show a dorsal view of each embryo for somite staining while panels F, J, and O show a dorsal view of *xESR9* expression. A GFP RNA injected embryo (500 pg) is shown in panels C, D, E, and F. *xBmal1* RNA injected embryos (500 pg) are shown in panels G, H, I, and J. *xNocturnin* RNA (500 pg) injected embryos is shown in panels K, L, M, and O. Black arrowheads show an example where the posterior *xESR9* stripes were aligned (F) or not aligned (J, O) between the injected and uninjected sides.

**Figure 6:**
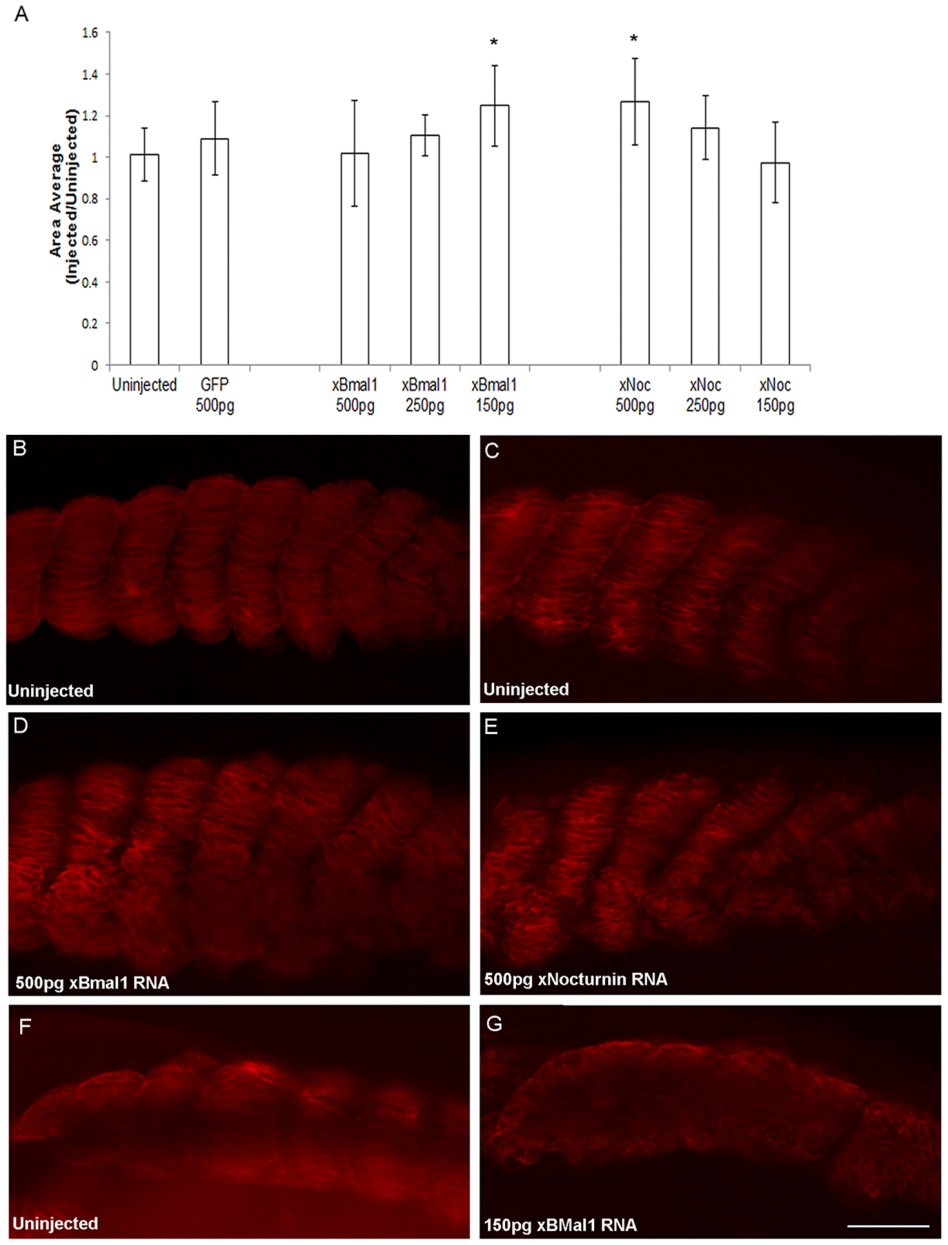
Overexpression of xBMAL1 or xNOCTURNIN protein results in larger, disorganized somites with disrupted somite boundaries. Panel A represents the results of analyzing paired somite area (uninjected side vs injected) using NIH Image J. The average ratio of the area of injected to uninjected sides of the embryos analyzed is shown with error bars representing standard deviation. A significant difference in area between 150 pg *xBmal1* RNA injection (*; p<0.02) and 500 pg *xNocturnin* RNA injection was observed (*; ANOVA posthoc LSD compared to control MO, p<0.009). Panels B-G compare muscle actin (12/101) of the uninjected (B, C, F) and injected sides (D, E, G) of embryos overexpressing xBMAL1 (D, 500 pg; G, 150 pg) or xNOCTURNIN (E, 500 pg). All images were taken at the same magnification (scale bar = 10 µm).

Surprisingly, overexpression of xBMAL1 and xNOCTURNIN protein decreased the number of somites formed in the early embryo. We had expected to see the opposite result based on the results of our MO experiments. One cell of two celled embryos was injected with *GFP* (500 pg), *xBmal1* (500 pg, 250 pg, 150 pg) or *xNocturnin* (500 pg, 250 pg, 150 pg) mRNA. Embryos were cultured to stage 25–28 and analyzed for *xESR9* and muscle actin as in the experiments outlined above. Overexpression of xBMAL1 or xNOCTURNIN protein caused a significant decrease in somite number when compared to GFP injected embryos (Figure 5A, Table 3; ANOVA p<0.001; posthoc LSD p<0.009). Comparison of somite number on the injected and uninjected sides of embryos commonly yielded an equal number of somites when GFP was injected (Figure 5C (uninjected side), D (1 ng GFP), E (dorsal view)). Injection of *xBmal1* RNA (Figure 5 G–I; 500 pg) resulted in less somites present on the injected side. Similarly somite number was reduced with injection of *xNocturnin* RNA (Figure 5 K–M; 500 pg). The effect on somite number was dose dependent (Figure 5A; Table 3). Another striking phenotype was the effect of overexpression of either protein on somite organization and boundary formation. Embryos injected with *xBmal1* RNA displayed disrupted somite borders (Figure 6D). In some cases we again observed a lack of somite boundaries with *xBmal1* injection (Figure 6 F,G; 150 pg *xBmal1* RNA). Overexpression of xNOCTURNIN protein resulted in less distinct somite boundaries on the injected side as well (Figure 6E). The somites of embryos injected with *xBmal1* or *xNocturnin* RNA also looked larger. We again used NIH ImageJ to measure somite area [44]. ANOVA analysis (p<0.001) showed a significant difference in the average areas among experimental conditions. LSD post hoc testing found a significant increase in somite area only when the controls (uninjected embryos or 500 pg GFP) were compared to embryos treated with 150 pg *xBmal1* (p<0.02) or 500 pg *xNocturnin* RNA (p<0.006; Figure 6A).

**Table 3:** Effect of RNA injection on somite number in stage 24–28 embryos.

Somite Number (injected vs uninjected)	GFP (500 pg) N = 20	*xBmal1* (500 pg) N = 32	*xBmal1* (250 pg) N = 21	*xBmal1* (150 pg) N = 23	*xNoc* (500 pg) N = 22	*xNoc* (250 pg) N = 28	*xNoc* (150 pg) N = 20
Equal	80%	3.125%	14.3%	17.4%	18.2%	28.6%	25%
Decreased by 1–2 somites	10%	37.5%	52.4%	65.2%	45.4%	53.6%	60%
Decreased by less than 2	0%	53.125%	23.8%	13.1%	36.4%	14.3%	15%
Increased by 1–2 somites	10%	6.25%	9.5%	4.3%	0%	3.6%	0%
Increased by more than 2	0%	0%	0%	0%	0%	0%	0%

Expression of *xESR9* in embryos injected with *xBmal1* or *xNocturnin* mRNA was also affected. We found a significant effect on the number of posterior stripes and extent of tailbud expression of *xESR9* in embryos injected with all doses of *xBmal1* (500 pg, N = 18; 250 pg, N = 26; 150 pg; N = 23) or *xNocturnin* (500 pg, N = 28; 250 pg, N = 28; 150 pg, N = 18) when compared to *GFP* (500 pg; N = 26) using a Mann-Whitney U test (p<0.02; Figure 5B). Examples of *xESR9* expression in GFP injected (Figure 5F), *xBMal1* injected (Fig. 5J; 500 pg) and *xNocturnin* (Fig. 5O; 500 pg) injected embryos is shown. Altered numbers of stripes and posterior borders on the injected versus the uninjected sides is shown in Figure 5J and O (black arrowheads).

## Discussion

Work in mice, rats, zebrafish, and frog shows that the expression of circadian genes in an embryonic organ or tissue precedes the initiation of time of day dependent (circadian) expression of those genes [34], [45], [46], [47], [48], [49], [50], [51]. Interestingly, although *Bmal1* and *mPer2* are expressed, no detectable circadian rhythm is observed in mouse embryonic stem cells and iPS cells (*Bmal1*::LUC) or embryonic cells in primary culture (*mPer2*::LUC) until after differentiation factors are added [52], [53]. *xPeriod1, xPeriod2*, *xBmal1*, *xNocturnin, xCryptochrome 1, xCryptochrome 2*, and *xClock* are present in the early neural plate and developing eye before a detectable circadian rhythm is present [34], [54]. Therefore, it is possible that circadian genes play different roles in the developing embryo before and after organ differentiation. In this paper, we began to investigate interactions between known circadian clock components and somite differentiation.

### Circadian gene expression: The right time and place

All of the circadian genes that we have analyzed so far are expressed in the developing somites (Figure 1; Figure S1) [34]. We have analyzed the developmental expression of 6 central oscillator genes (*xPeriod1*, *xPeriod2, xBmal1*, *xClock*, *xCry1*, and *xCry2*) and one CCG (*xNocturnin*). We do not see circadian genes expressed in waves in the presomitic mesoderm as expected for components of the somite clock [9]. *xBmal1* and *xNocturnin* are present in the presomitic mesoderm and throughout the newly formed somite, but they continue to be expressed after the somites are formed (Figure S1) [34], suggesting their continued involvement in somite differentiation after segmentation. Microarray analysis has detected harmonics of the period of circadian gene expression in the developing somite (Table 1) [35] as well as in adult mouse liver and heart (8, 12, 24 hours) [55] suggesting that the temporal period of circadian gene expression may vary during development. Another strong candidate that may influence the somite clock is *xPeriod1* since it is expressed strongly in the stem cell population of the tailbud that gives rise to the presomitic mesoderm [34] similar to somite clock components like *xESR9* and *HES5* [9], [42], [56]. Future experiments will define the temporal expression of circadian genes such as, *xPeriod1*, during and after somite formation.

### Depletion and overexpression of xBMAL1 and xNOCTURNIN affects somite number

We were able to perturb somitogenesis by depleting or overexpressing two circadian genes. We were able to monitor the dosage effects of these genes by comparing endogenous somite formation or *xESR9* gene expression on one side of the embryo with the experimentally manipulated side. Depletion and overexpression of xBMAL1 or xNOCTURNIN protein resulted in similar effects suggesting that they may be acting in the same pathway. Morpholino injection, in general, was found to affect the number of somites present on the injected side (Figure 3; Figure S3). In *Xenopus*, Davis and colleagues [57] show that *hairy2a* expression on the right side of the presomitic mesoderm consistently precedes expression on the left side, which could explain some underlying asynchrony in somite formation in our experiments. Similar results have been reported for *xESR9* [42]. The sensitivity of somite formation to the control MO injection was dose dependent as shown in the Figure S3 (500 pg and 1.5 ng) but in almost all cases *xBmal1* MO and *xNocturnin* MO injection resulted in fewer somites forming on the injected side while the control MO effects were more variable. Also, the effect of injection of our circadian gene MOs was significantly different from the control (p<0.006 and p<.001, respectively). Therefore, the overall effect of *xBmal1* MO and *xNocturnin* MO injection is specific. We also showed that injection of experimental MOs could decrease the concentration of xBMAL1 and xNOCTURNIN protein in the embryo when compared to the control MO (Figure 2). The *xNocturnin* MO did not decrease xNOCTURNIN protein when compared to the *xBmal1* MO which may explain the less dramatic effects of *xNocturnin* MO in our experiments. Interestingly, embryological defects have not been reported for *Nocturnin* knock-out mice although *Bmal1* KO mice are not very healthy and show skeletal muscle defects (a somite derivative) [40], [41].

### Circadian genes influence somite differentiation and myogenesis

**Figure 7:**
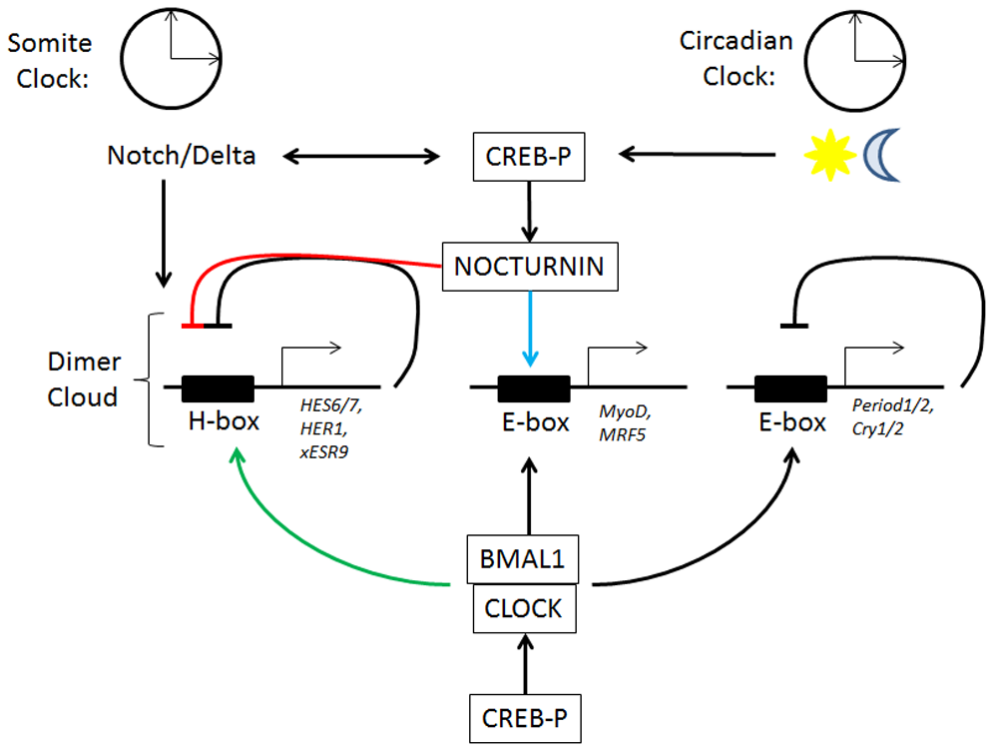
Here we speculate on the possible interactions between cell autonomous circadian and somite oscillator components during somite formation and differentiation. The somite and circadian clocks both consist of negative feedback loops requiring the transcriptional activation (BMAL1, CLOCK) and repression (HES6/7, HER1; PERIOD1/2, CRY1/2) of clock components. The period of the somite clock is thought to be regulated by the balance of bHLH transcription factors present in the cell (“dimer cloud”) [13]. Bmal1 may upset the balance by hetero-dimerizing with proteins in the dimer cloud (HES6/7, HER1) or by competing with HES6/7/HER1 for binding to the H-box (green arrow). NOCTURNIN may also impact the balance of proteins present in the dimer cloud by inhibiting translation of dimer cloud components (red line). BMAL1-CLOCK heterodimers are known to positively activate genes involved in myogenesis (*MyoD*, *MRF5*). Perhaps, NOCTURNIN inhibits translation of repressors of myogenesis (blue arrow). We hypothesize that phosphorylation of CREB protein (CREB-P) may also act to coordinate components of the circadian and somite clock.

The effect of depletion and overexpression of xBMAL1 on somite size suggests that xBMAL1 positively influences somite size and therefore myogenesis. The marker we used in our experiments (12/101) is a generally accepted marker of muscle formation in the embryo [43]. Circadian expression of *MyoD* in adult skeletal muscle, a somite derived tissue, is regulated by BMAL1 and mice deficient in *Bmal1* or its binding partner (*Clock*) have decreased skeletal muscle [40], [41]. More recently, researchers have shown BMAL1 controls myogenesis in cultured C2C12 myoblasts through WNT signaling [58]. We think that circadian genes may also play a role in embryonic myogenesis since they continue to be expressed in the developing somite after the somite clock has stopped. BMAL-CLOCK heterodimers regulate circadian expression of *MyoD* in adult skeletal muscle through a non-canonical E-box (CAGCTT) [40], [41], [59]. Adult mice deficient in BMAL1 display disorganization of myofibrils and decreased force of contraction in both skeletal and cardiac muscle [41], [60]. Expression of myogenic genes (*Myf5*, *Mrf4*, and *MyoD*) was decreased in *Bmal* deficient C2C12 myoblasts cells and upregulated when *Bmal1* was overexpressed [58]. We report similar effects of depletion and overexpression of *xBMAL1*, supporting the interpretation that xBMAL1 is likely a positive regulator of embryonic myogenesis (Figure 7).

Results from depletion and overexpression of xNOCTURNIN also suggest a positive influence on somite size and myogenesis. xNOCTURNIN is a deadenylase and thus acts as a post-transcriptional regulator [29]. Depletion of xNOCTURNIN decreased somite size, while increasing doses caused somite size to increase suggesting that xNOCTURNIN may act to repress translation of inhibitors of somite differentiation and myogenesis. *Dec1, Dec 2* and *Id* are circadianly expressed bHLH transcription repressors that have been shown to repress BMAL1/CLOCK function and myogenesis [61], [62], [63], [64], [65]. If xNOCTURNIN does inhibit translation of genes like *Dec1/2* and *Id* then we would expect overexpression of xNOCTURNIN to result in larger somites and depletion of xNOCTURNIN to result in smaller somites. To date, NOCTURNIN has been implicated in the regulation of sugar and fat metabolism as well as adipogenesis in adult mice [31], [66]. In the mouse embryo, *Nocturnin* has been shown to regulate RNA stability during maternal to zygotic transition [67]. Therefore, our results suggest a new function for this protein in somite differentiation and myogenesis.

Overexpression of xBMAL1 and xNOCTURNIN caused an increase in somite area. One puzzling aspect of these results is that only the lowest dose (150 pg) of *xBmal1* RNA significantly increased somite area. xBMAL1 is a bHLH transcriptional coactivator that coordinates with CLOCK to bind to the E-box of circadianly regulated genes such as *MyoD* [21], [22], [23], [40], [41]. xBMAL1 function requires binding with other bHLH transcription factors (such as CLOCK) which are likely limiting or even negatively impacting the effect of higher concentrations of xBMAL1 on somite size.

Somite size and boundary formation can also be affected by Wnt signaling [12], [68]. A recent report shows that inhibition of WNT signaling can increase the velocity of the wavefront and thus increase somite length [68]. GSK3 activity is a possible link between WNT signaling and the circadian oscillator (Table 1). Although intriguing, it is difficult to reconcile our results with the role of wavefront velocity in somite size and boundary formation at this time.

### Circadian genes influence the somite clock

Manipulating the dosage of xBMAL1 and xNOCTURNIN caused somites to appear disorganized and lack distinct boundaries. In some cases, both depletion and overexpression of either gene product can eliminate somite boundaries altogether forming a “streak” of muscle actin on the side of the embryo (Figure 4 and 6). We also see an effect on the posterior striping pattern of *xESR9* indicating an effect on a component of the somite clock. The somite clock determines when somites will form from the presomitic mesoderm and has been implicated in boundary formation between individual somites. Perturbation of members of the Notch signal transduction pathway (specifically *Notch1*, *Delta-like 1*, *HES1, 5, 7* and *Hey1*) lead to improper segmentation and disorganized somites [69], [70], [71], [72], [73], [74], [75]. We observed similar effects on somite organization while perturbing the levels of xBMAL1 and xNOCTURNIN protein levels suggesting that these genes may modulate somite clock function.

If manipulation of circadian gene dosage is slowing the period of oscillation of the somite clock, then we would predict that fewer but larger somites would form [9], [13], [14]. Both depletion and overexpression of xBMAL1 and xNOCTURNIN led to fewer somites, but had opposite effects on somite size (Figures 4 and 6). Recently, Dias and colleagues [16] reported that somite formation can occur in the absence of a detectable somite clock, suggesting that the processes of placement and timing of somites is separable from differentiation. Therefore, it is possible that *xBmal1* and *xNocturnin* modulate oscillations of the somite clock in the presomitic mesoderm and later play a role in somite differentiation.

### Possible interactions between somite and circadian clock components

Oscillations of both circadian and somite genes are thought to be controlled by negative transcriptional/translational feedback loops. Possible points of interaction between the somite clock and circadian clock include heterodimerization of bHLH transcription factors, competition for binding to promoters of clock regulated genes, and CREB phosphorylation. The model proposed in Figure 7 is highly speculative and highlights possible interactions between circadian genes and somite clock components providing a framework that will inform future experiments.

Depletion and overexpression of xBMAL1 and xNOCTURNIN may upset the balance of bHLH proteins present in the cell leading to slowing of the somite clock (Figure 7). Recent evidence suggests that somite clock gene products bind to the promoters of cyclically controlled genes at specific sites termed H-boxes (CACGNG with N = T preferred) [13]. The balance of homo and hetero-dimers control the molecular oscillations in gene expression in core components of the somite clock. The H-box is similar in sequence to canonical E-boxes found in promoters regulated by circadian clock gene products, BMAL-CLOCK heterodimers (CACGTG and CACGTT) [19]. Since xBMAL1 is a bHLH transcriptional activator it may form heterodimers with HER1, HER7, or HES6 directly and influence their ability to repress transcription of core somite clock components. Alternatively, xBMAL1/CLOCK heterodimers may compete for binding to H/E boxes of somite clock controlled genes. Since the dosage of NOCTURNIN protein yields similar phenotypes on somite formation to that of xBMAL1 it may be that NOCTURNIN also regulates the proportion of bHLH transcription factors present in a cell (Figure 7). Another link between *Nocturnin* regulation by the circadian clock and somite clock may be phosphorylation of CREB (CREB-P). CREB-P has been shown to positively influence the expression of myogenic genes (*Myod*, *MyF*5), *xNocturnin*, entrainment of the circadian clock, and is cyclically expressed in the presomitic mesoderm [76], [77], [78], [79], [80].

## Materials and Methods

### Animal Care and Embryo collection

Animal care and experiments performed in the manuscript were approved by the University of Wisconsin Animal Care and Use Committtee (Animal Study Protocol #C11207002Q). Pigmented *Xenopus laevis* (NASCO) was used for all experiments. Eggs were collected from females injected with 800 units of human chorionic gonadotropin (Patterson Veterinary Supply, Inc.) and fertilized with macerated testis. Embryos were then maintained in a low ionic strength salt solution, 1/3X Modified Barth's Solution (MBS) [81]. All embryo stages were determined according to Nieuwkoop and Faber [2].

### Embryo injection and culture

Embryos were injected at the two cell stage in 1X MR (solution components) containing 2–3% Ficoll. For analysis of the effects of injection on somite number, area, and *xESR9* expression, one cell of a two cell stage embryo was injected with 10 nl of solution using a Sutter Instruments Xenoworks Digital Microinjector. We used fluorescently labeled morpholinos designed by GeneTools that blocked the translation start site for *xNocturnin* (GTGAGCTGTGCATCCATTCTACCTG) and *xBmal1* (GCCATTGGATCATCGTCAGGCGCAC). The GENETOOLS fluorescein labeled control morpholino was used as a control. In vitro transcription was performed using a clone of *xBmal1* (pBSxBmal1-a, 2.8 kb full length in pBS-KS+) and *xNocturnin* (CS2+) kindly provided by Dr. Carla Green. *GFP*-CS2+ was kindly provided by Dr. Michael Zuber. The pBS*xBmal1-a* clone was sub-cloned into a CS2+ vector using standard procedures and sequenced to confirm the proper orientation of the clone in the vector. In vitro transcription of linearized (*Not1*) *xBMal1*-CS2+, *xNocturnin*-CS2+, *GFP*-CS2+ was performed using Riboprobe system SP6 (Promega P1420). RNA levels were quantified using UV spectrophotometry (Nanodrop 2000c) and gel electrophoresis. We included 0.1% FLDX in all injection solutions to make it easier to see the solution while injecting embryos. If RNA was present in the solution we added RNAsin to the 1% FLDX stock solution (therefore approximately 0.67 u/µl of RNAsin was present in injection solution). Injection of embryos with experimental MOs or mRNA typically caused exogastrulation (20–32%) and death (12–50%) of injected embryos. A range of 18–50% of injected embryos per experiment exhibited relatively normal development in the posterior with clearly defined injection of the right or left side. These are the embryos we used in subsequent analyses. We did not analyze exogastrulated embryos since it would likely affect *xESR9* expression and our interpretation of somite number. Embryos with mild exogastrulation were analyzed, but if the exogastrulation hampered our ability to discriminate the posterior striping pattern of *xESR9* they were designated as uninterpretable.

### In situ hybridization

Stage 25–28 embryos were fixed in MEMFA (0.1M MOPS pH 7.4, 2 mM EGTA pH 8, 1 mM MgSO_4_, 3.7% formaldehyde) for one hour, washed in methanol, and stored in methanol at −20°C. In situ hybridization was carried out according to the procedure of Harland [82] as modified by Doniach and Musci [83]. Clones for in situ probes were kindly provided by Dr. Carla Green. All embryos were bleached after probe hybridization and SSC washes and before the antibody blocking steps so that the subsequent color reaction could be easily monitored.

### Immunohistochemistry

Following in situ hybridization, embryos were briefly refixed in MEMFA for 30 minutes and then analyzed using immunohistochemistry. The protocol was based on the procedure of Brivanlou and Harland [84]. The primary antibody used was 12/101 (a muscle-specific antibody that is used as a somite marker) [44] and was used at a dilution of 1∶10. The secondary antibody, Red Fluorescent AlexaFLuor 568 antimouse IgG (Invitrogen, A21124), was diluted 1∶400.

### Western blot

Two celled embryos were injected with 500 pg or 1 ng of *xBmal1* or *xNocturnin* MO in each cell (total of 1–2 ng total MO/embryo). Embryos were then cultured to stage 25–28, checked for the presence of fluorescein, and quick frozen in dry ice, based on protocol by Baggs and Green [29]. The protein concentration was determined for each sample using Bradford assay (BioRad). The volume of sample per tube was adjusted for equal loading of protein per well (approximately 20 µg/well). Blots were incubated overnight at 4°C with a 1∶500 dilution of either xNocturnin antibody [28], Goat anti-Bmal1 (ARNTL) antibody (MyBioSource MBS422067), or anti-alpha A2 (DM1) tubulin (Neomarkers MS-581-P0). The polyclonal anti-Bmal1 antibody was made using an internal consensus region of the human Bmal1 protein. Comparison of the peptide sequence used was different from the *Xenopus* protein sequence at amino acid 409 (F→Y; Blast Sequence Comparison). A band of the expected protein size of *Xenopus* xBMAL1 (69 kd) was recognized using this antibody showing that the antibody recognizes the *Xenopus* protein. Membranes were washed three times for ten minutes with TBST (20 mM Tris, pH 7.6, 150 mM NaCl, .1% Tween) and incubated with 1∶4000 dilution of peroxidase conjugated Goat anti rabbit-HRP (Promega W4011; recognizes Nocturnin primary), Bovine anti Goat IgG-HRP (Santa Cruz Biotechnology; sc2350; recognizes Bmal1 primary) or goat anti-mouse IgG (H+L) (Jackson Immunological Laboratory; recognizes Tubulin primary) for one hour at room temperature. Following washes as above, additional washes in TBST(0.3% Triton) and TBS were performed before development with chemiluminescent reagents (Thermoscientific-supersignal substrate; #34075). Western blot analysis was performed 3 times with similar results in protein depletion for both *xBmal1*-MO and *xNocturnin*-MO treatments.

Quantification of the relative amounts of xBMAL1 and xNOCTURNIN protein in control vs MO injected embryos was done using NIH Image J [44]. The intensity of each band was measured using NIH Image J. The relative amount of protein in each lane with respect to control MO injected embryos was calculated by first determining the density of each band with respect to the control MO injection. Next, the relative densities of each sample were normalized to the relative density of the loading control (alpha tubulin) in each lane.

### Photography

Images of whole embryos were taken using an Olympus MVX10 stereoscope with Olympus DP70 digital camera. Images in figures 4 and 6 were taken in cleared embryos (BB/BA) using an Olympus IX2-DSU disk scanning microscope with a Hamammatsu ORCA-ER digital camera. Adobe Photoshop CC was used to assemble and modify all figures.

### Embryo scoring and statistics

Before fixation all stage 25–28 embryos were evaluated using fluorescence microscopy (presence of fluorescein tag on MO or in RNA) to determine which side of the embryo was injected with a specific treatment. Uninjected embryos, embryos with fluorescent labeling on both sides, and embryos that experienced severe exogastrulation were not analyzed. The number of somites present on the injected and uninjected sides of each embryo was recorded. We then calculated the difference in somite number between the injected and uninjected side of each embryo. Differences in somite number for each treatment condition were compared using ANOVA and LSD posthoc test (IBM SPSS20). The data conformed to a normal distribution and the LSD posthoc test was used to compare the average number of somites found in the control injected embryos with the average number of somites found in morpholino or RNA injected embryos.

Similarly, each embryo was scored for *xESR9* expression on the injected and uninjected side. The number of posterior stripes was assessed on each side (0–3 stripes) as well as any effects on the border of the posterior band of expression of *xESR9* in the presomitic mesoderm. Next, the score for the injected side of each embryo was subtracted from the score of the uninjected side. A result of zero indicated that the pattern of expression was similar between the injected and uninjected sides of the embryo. A positive or negative number indicated that there was more or less stripes of *xESR9*, respectively, present in the injected side when compared to the uninjected side. We used the absolute value of the calculated difference to compare treatment conditions (control vs experimental). These data were assessed nonparametrically using a Mann-Whitney-U test.

Lastly, we took pictures of the left and right sides of embryos injected with MO or RNA. We used NIH ImageJ to calculate the area of 3 consecutive pairs of somites per embryo using (usually somites 3–5, anterior-posterior) [44]. The difference in area between each pair of somites (injected vs uninjected) was calculated. The average difference in area for 3 somites was then recorded for each embryo. The average difference in area measurements were compiled for each treatment (control morpholino, *xBmal1* MO, etc). The treatment results were normally distributed and were first compared using ANOVA. Next each experimental average was compared to its associated control (i.e. control MO vs *xBmal1* 1MO) using a LSD posthoc test (IBM SPSS20).

## Supporting Information

Figure S1 **Circadian genes are expressed in the somites during tailbud stages.** Co-localization of the mRNA expression and 12/101 protein (somite marker) are shown in each pair of panels, such as A and A′. The white dotted lines were drawn on the borders of the in situ expression pattern for each gene and positioned in the exact same position over the 12/101 expression. In all cases the circadian genes were present throughout the somite and excluded from the myocoel.
(TIF)

Figure S2 **A summary of the developmental expression of** ***xClock*** **,** ***xCry1*** **, and** ***xCry2*** **.** In situ hybridization was performed on stage 35–38 embryos. Dorsal view of the head and lateral views of the entire embryo are shown for *xClock* (A,B), *xCry1* (C,D), and *xCry2* (E,F). Black arrowheads highlight the otic vesicle while white arrows show expression in the olfactory bulb. Red arrowheads highlight the pronephric tubules while red arrows show the pronephric duct if visible. The cement gland is indicated by a green arrowhead. A developmental time series is provided (G) below the images to show the earliest we were able to detect each gene's expression in various embryonic organs and tissues.
(TIF)

Figure S3 **Depletion of xBMAL1 or xNOCTURNIN results in fewer somites on the injected side.** The percent of embryos with equal, less, or more somites on the injected side when compared to the uninjected side is indicated on the vertical axis. The concentration and type of MO injected is shown on the horizontal axis. Injection of 500 pg of xBmal1 MO (N = 54) consistently resulted in fewer somites when compared to control MO Injection (500 pg; N = 26). Injection of 1.5 ng of xNocturnin MO (N = 33) consistently resulted in fewer somites when compared to the more variable phenotype displayed by control MO injection (N = 30).
(TIF)

Figure S4 **In some cases, depletion of xBMAL1 and xNOCTURNIN protein affected** ***xESR9*** **expression in the developing eye and central nervous system.** In panel A, depletion of xBMAL1 protein (500 pg xBmal1MO injection; *) decreased expression of *xESR9* in the eye (white arrowhead). Comparison of the width of *xESR9* expression in the hindbrain and spinal cord shows a wider expression of *xESR9* on the injected side, indicated by the width of the white line, when compared to the uninjected side (width of black line). Panels B and C show the effects of depletion of xNOCTURNIN (1 ng, *). Depletion of xNOCTURNIN decreased expression of *xESR9* in the eye (white arrow head) and decreased *xESR9* expression in the brain and spinal cord. The embryo in panel C was also anencephalic.
(TIF)
