# Supplementary figures and images for: Circadian Genes, xBmal1 and xNocturnin, Modulate the Timing and Differentiation of Somites in Xenopus laevis
Source: PLoS One. 2014 Sep 19;9(9):e108266. doi: 10.1371/journal.pone.0108266 (PMC4169625; doi:10.1371/journal.pone.0108266)

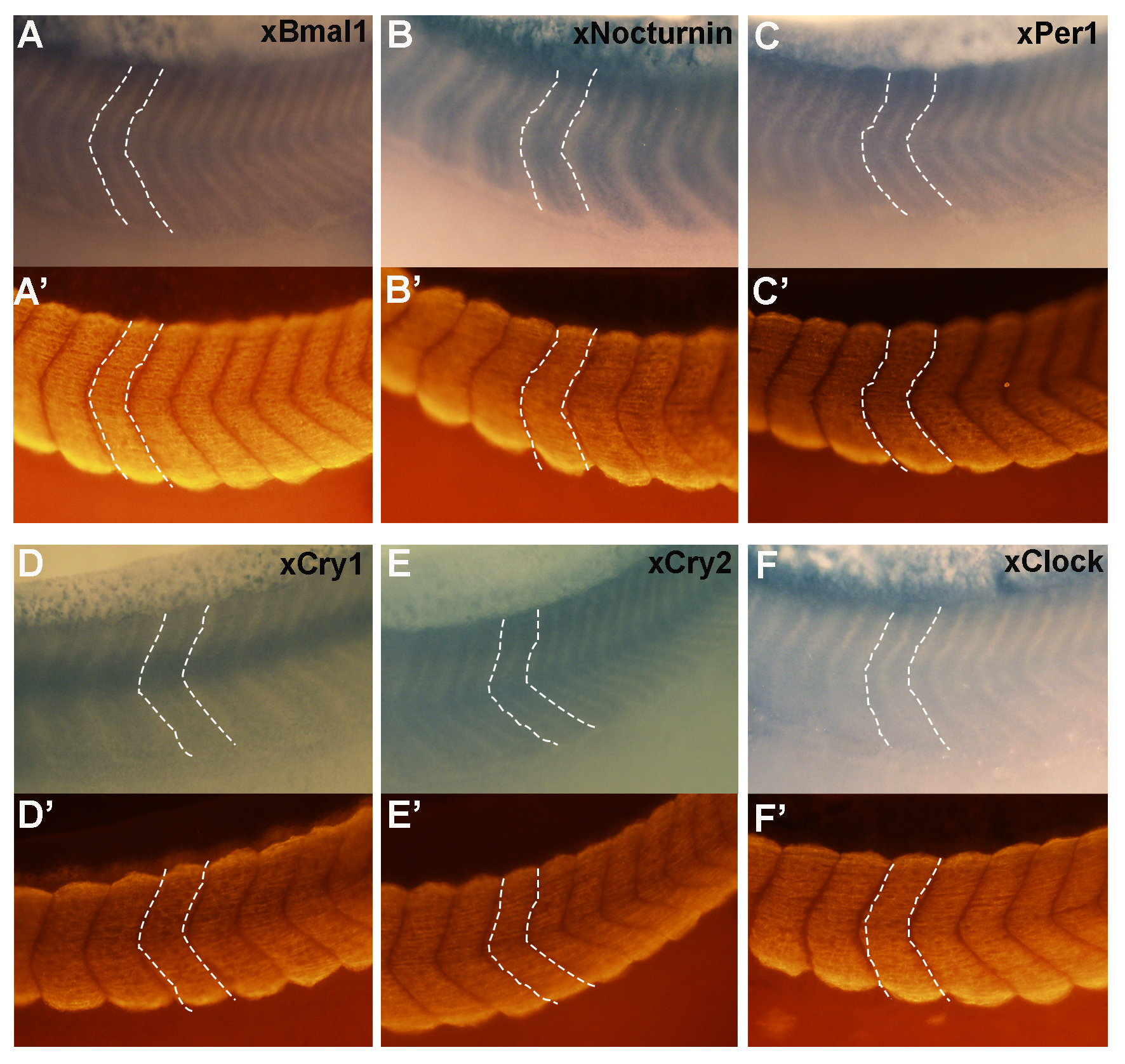

Supplement: Figure S1 — Circadian genes are expressed in the somites during tailbud stages. Co-localization of the mRNA expression and 12/101 protein (somite marker) are shown in each pair of panels, such as A and A′. The white dotted lines were drawn on the borders of the in situ expression pattern for each gene and positioned in the exact same position over the 12/101 expression. In all cases the circadian genes were present throughout the somite and excluded from the myocoel. (TIF) [file pone.0108266.s001.tif]

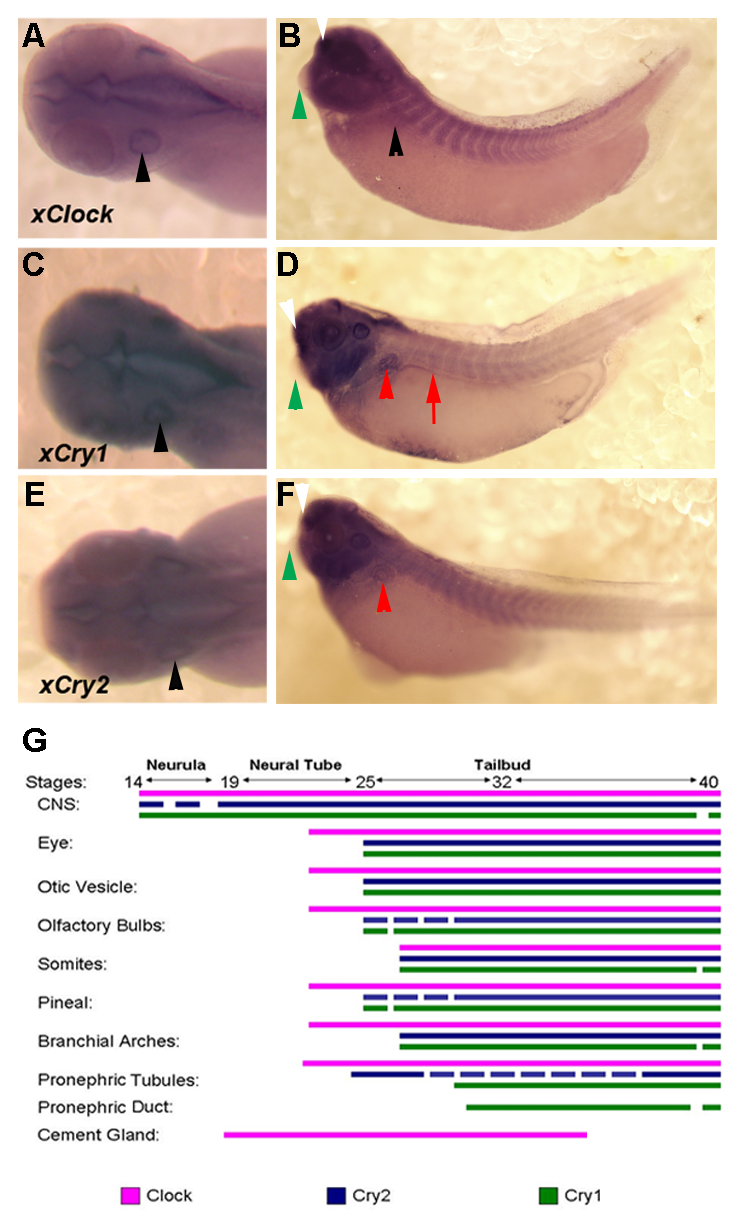

Supplement: Figure S2 — A summary of the developmental expression of xClock , xCry1 , and xCry2 . In situ hybridization was performed on stage 35–38 embryos. Dorsal view of the head and lateral views of the entire embryo are shown for xClock (A,B), xCry1 (C,D), and xCry2 (E,F). Black arrowheads highlight the otic vesicle while white arrows show expression in the olfactory bulb. Red arrowheads highlight the pronephric tubules while red arrows show the pronephric duct if visible. The cement gland is indicated by a green arrowhead. A developmental time series is provided (G) below the images to show the earliest we were able to detect each gene's expression in various embryonic organs and tissues. (TIF) [file pone.0108266.s002.tif]

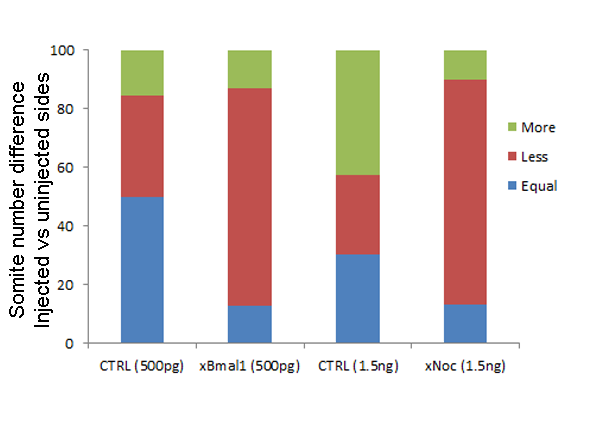

Supplement: Figure S3 — Depletion of xBMAL1 or xNOCTURNIN results in fewer somites on the injected side. The percent of embryos with equal, less, or more somites on the injected side when compared to the uninjected side is indicated on the vertical axis. The concentration and type of MO injected is shown on the horizontal axis. Injection of 500 pg of xBmal1 MO (N = 54) consistently resulted in fewer somites when compared to control MO Injection (500 pg; N = 26). Injection of 1.5 ng of xNocturnin MO (N = 33) consistently resulted in fewer somites when compared to the more variable phenotype displayed by control MO injection (N = 30). (TIF) [file pone.0108266.s003.tif]

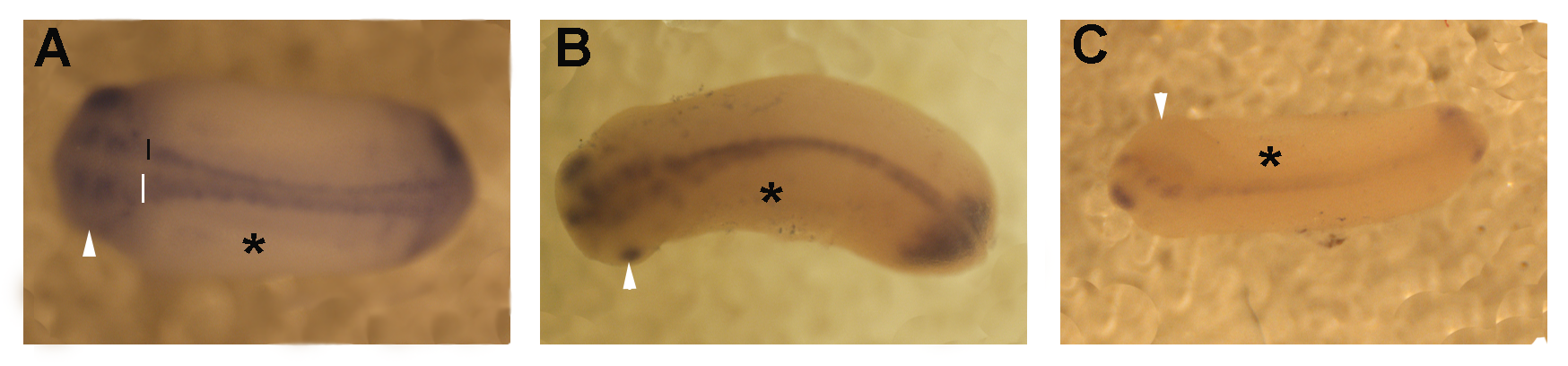

Supplement: Figure S4 — In some cases, depletion of xBMAL1 and xNOCTURNIN protein affected xESR9 expression in the developing eye and central nervous system. In panel A, depletion of xBMAL1 protein (500 pg xBmal1MO injection; *) decreased expression of xESR9 in the eye (white arrowhead). Comparison of the width of xESR9 expression in the hindbrain and spinal cord shows a wider expression of xESR9 on the injected side, indicated by the width of the white line, when compared to the uninjected side (width of black line). Panels B and C show the effects of depletion of xNOCTURNIN (1 ng, *). Depletion of xNOCTURNIN decreased expression of xESR9 in the eye (white arrow head) and decreased xESR9 expression in the brain and spinal cord. The embryo in panel C was also anencephalic. (TIF) [file pone.0108266.s004.tif]
